# Gender Difference of Unconscious Attentional Bias in High Trait Anxiety Individuals

**DOI:** 10.1371/journal.pone.0020305

**Published:** 2011-05-24

**Authors:** Jieqing Tan, Zheng Ma, Xiaochao Gao, Yanhong Wu, Fang Fang

**Affiliations:** 1 Department of Psychology, Peking University, Beijing, China; 2 Learning and Cognition Lab, Capital Normal University, Beijing, China; 3 Yuanpei College, Peking University, Beijing, China; The University of Queensland, Australia

## Abstract

By combining binocular suppression technique and a probe detection paradigm, we investigated attentional bias to invisible stimuli and its gender difference in both high trait anxiety (HTA) and low trait anxiety (LTA) individuals. As an attentional cue, happy or fearful face pictures were presented to HTAs and LTAs for 800 ms either consciously or unconsciously (through binocular suppression). Participants were asked to judge the orientation of a gabor patch following the face pictures. Their performance was used to measure attentional effect induced by the cue. We found gender differences of attentional effect only in the unconscious condition with HTAs. Female HTAs exhibited difficulty in disengaging attention from the location where fearful faces were presented, while male HTAs showed attentional avoidance of it. Our results suggested that the failure to find attentional avoidance of threatening stimuli in many previous studies might be attributed to consciously presented stimuli and data analysis regardless of participants' gender. These findings also contributed to our understanding of gender difference in anxiety disorder.

## Introduction

Generalized anxiety disorder (GAD) is an anxiety disorder that is characterized by excessive, uncontrollable and often irrational worry about everyday things, which is disproportionate to the actual source of worry [1]. To study its psychopathology, researchers usually adopted patients with generalized anxiety disorder as clinical sample and individuals with high trait anxiety as subclinical sample [2]. Recently, studying subclinical or non clinical population was recommended for the convenient participant recruitment and the exclusion of factors of medicine and therapy.

Cognitive theories about generalized anxiety disorders propose that patients or HTA individuals have cognitive vulnerabilities at the level of attentive processing of threat that may maintain anxiety, and may even lead to the development of clinical anxiety disorders [3], [4]. Several studies [5], [6] have suggested that the attentional system of anxious individuals may be abnormally sensitive to threat-related stimuli in the environment, leading to an even more pronounced processing bias in favor of threat-related stimulation than is observed in non-anxious individuals. The role of the attentional bias played in the development and maintenance of anxious disorders has been studied for about two decades [7]. Mogg and Bradley [8] proposed the “vigilance-avoidance” pattern to interpret the cognitive processing in anxious populations. HTA individuals initially attend to threat, but this is often followed by attentional avoidance of threat. This pattern of vigilance and avoidance is hypothesized to maintain anxiety [9].

Researchers usually used a dot-probe detection paradigm [10] to investigate the attentional bias in high trait anxiety population. In this paradigm, participants were exposed to a word pair or a picture pair on a computer screen, which included one threatening and one neutral word/picture. After the exposure, a dot (the probe) appeared in the location of one of the words/pictures. Participants were instructed to press a button as fast as possible to indicate the detection of the probe. For a short presentation of the stimulus pair (i.e. 500 ms), anxious participants were faster or more accurate to detect the probe when it was in the location of the threatening stimulus [5], [11], [12]. They exhibited attentional vigilance towards threatening stimuli. However, for a long presentation of the stimulus pair (i.e. 1250 ms or 1500 ms), no attentional effect was found in both HTA and LTA groups [5], [12]. This is not consistent with the “vigilance-avoidance” pattern proposed by Mogg and Bradley [8] because they predicted attentional avoidance of threatening stimuli with a long presentation. There are two potential reasons to explain the absence of attentional avoidance in previous studies: consciousness manipulation and gender difference in anxiety disorders. Our study aimed to address these two issues.

Many studies have demonstrated that attentional bias could be induced by an unconsciously presented cue [13]. For example, emotional Stroop task with backward masking was widely used in this field and researchers consistently found that HTAs exhibit attentional bias to threatening materials at subconscious level [14]–[16]. However, one drawback of backward masking is that this technique cannot render a stimulus invisible for a long presentation, thus is not suitable for test the “vigilance-avoidance” proposal.

This drawback can be overcome by another psychophysical method – binocular rivalry. When two incompatible pictures are presented to the two eyes that cannot be merged to a single visual percept, binocular rivalry occurs. Observer's perception switches back and forth between the two incompatible pictures, that is, they compete for perceptual dominance [17]. Some factors could boost the strength of one rival picture over another, such as high-contrast, brighter stimulus, moving contours, densely contoured, and stimuli presented in dominant eye [18]. Accordingly, the ‘stronger’ competitor enjoys an advantage in overall perceptual dominance. Jiang et al. [19] took advantage of binocular rivalry to study the effect of invisible images on the distribution of spatial attention. In their study, high contrast dynamic noise was presented to the dominant eye, and a meaningful picture was presented to the non-dominant eye. Because of the strong inter-ocular suppression by the dynamic noise, subjects were completely unaware of the meaningful picture. They found that a 800 ms presentation of invisible pictures could result in attentional bias and the bias was dependent on subjects' gender. This experimental paradigm is also call binocular suppression because of the imbalance of the strength of the two competing stimuli. In our study, we will use binocular suppression to render images invisible for a long presentation and investigate attentional bias at unconscious level.

We suspect that the failure to find attentional avoidance of threatening stimuli in many previous studies might be, at least partially, attributed to data analysis regardless of participants' gender. Many researches have indicated that there are gender differences in attention to and appraising of threat, which means females are more sensitive to threat-related cues than males and tend to overestimate the level of danger [20], [21]. McClure et al. [22] also found that orbitofrontal cortex and amygdala were selectively activated to unambiguous threatening stimuli in adult women but not men. What's more, some researches interested in gender differences in anxiety disorders [23]–[25] have shown that female suffer anxiety disorder much more frequently than male [11], [26], [27]. Waters and Valvoi [28] also proposed that there might be different ways for anxious and non-anxious girls to regulate their attention towards threatening faces. Thus, we proposed that there might be gender differences in the attentional bias to threatening cues and taking this variable into account may help us reconsider the “vigilance-avoidance” proposal. However, there was almost no study investigating the gender difference of anxious population with a cognitive approach. Only some researches in neurotic and high-defensive population had made such attempts. For example, the studies by Osorio et al. [29] and Jansson et al. [30] revealed that the relationship between neuroticism or defensiveness and attentional bias is affected by gender. It is likely that some gender effect on attentional bias may occur in high trait anxiety individuals.

In addition, many previous studies on attentional bias used an unbalanced gender ratio. For example, Koster et al. [7] recruited high trait anxiety participants with a gender ratio of female to male as 16 ∶ 6. Another ERP study about anxious individuals' perception of emotional faces used 8 female and 2 male [31]. The majority of female in researches might have led to a biased conclusion for the overall anxious population. The other aim of our study is to clarify the gender difference issue in attentional bias in high trait anxiety individuals, which has been ignored previously.

In summary, the main object of our study is to examine attentional bias and its gender difference at unconscious level for both HTA and LTA individuals. We predict that, at unconscious level, only HTA females might exhibit an attentional bias towards fearful faces.

## Experiment 1

### Methods

#### Participants

The experiment was conducted in accordance with general ethical guidelines in psychology. We have obtained written informed consent from all participants and the process was approved by the ethics committee in Peking University. In Experiment 1, we used a sub-clinical sample in order to avoid the effects of medical, educational and other related factors [2]. Participants were recruited from a pool of 1200 college students at Peking University according to their scores on the State-Trait Anxiety Inventory-Trait Anxiety sub-Inventory (STAI-TAI [32]; Chinese version by Zhen, et al., [33]). Those with a top 5% score were selected as High Trait Anxiety participants (HTAs) and those with a bottom 5% score were selected as Low Trait Anxiety participants (LTAs). A total of 24 HTAs and 24 LTAs, each including 12 female and 12 male respectively, participated in the experiment. Their STAI-TAI scores were in Table 1. The range of their age was 19–26 years, and they were naïve to the purpose of the study.

**Table 1:** STAI-TAI scores of female and male participants in HTA and LTA groups and T-Tests between these two groups.

	HTAMean (SD)	LTAMean (SD)	df	t	P
Female	56.67(11.18)	28.58(7.08)	22	7.35	<0.001
Male	58.50(11.29)	26.33(9.32)	22	7.61	<0.001

#### Stimuli and Procedure

Thirty-two images were selected from the Chinese Affective Picture System (CAPS) [34]. The images fell into four categories (fearful female, happy female, fearful male, happy male), eight images for each category. The contrast of images was adjusted to a low level to guarantee the effectiveness of binocular suppression (see below).

**Figure 1:**
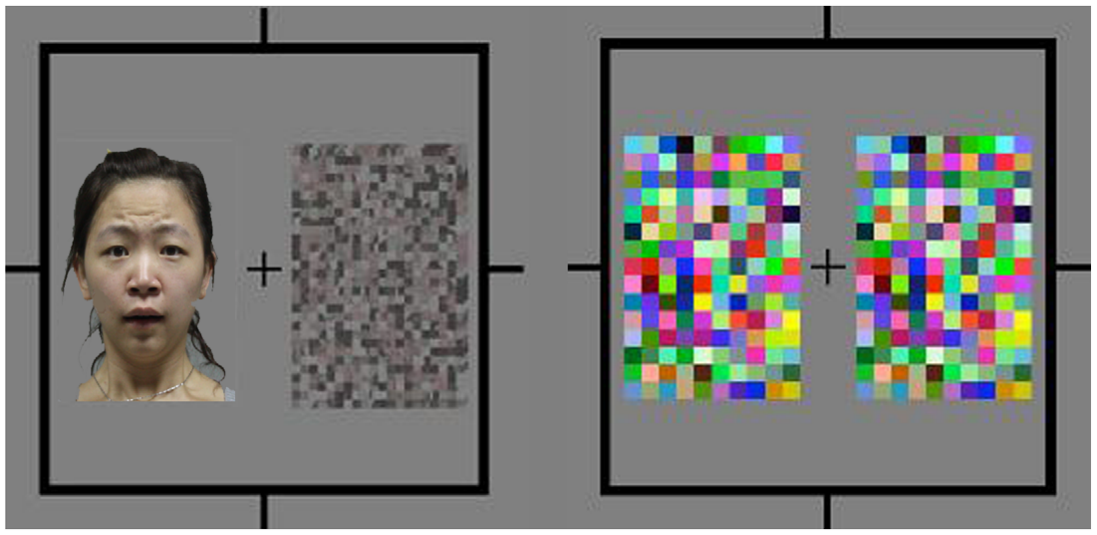
A sample stimulus in the invisible condition. The left image was presented to the non-dominant eye and the right image was presented to the dominant eye.

**Figure 2:**
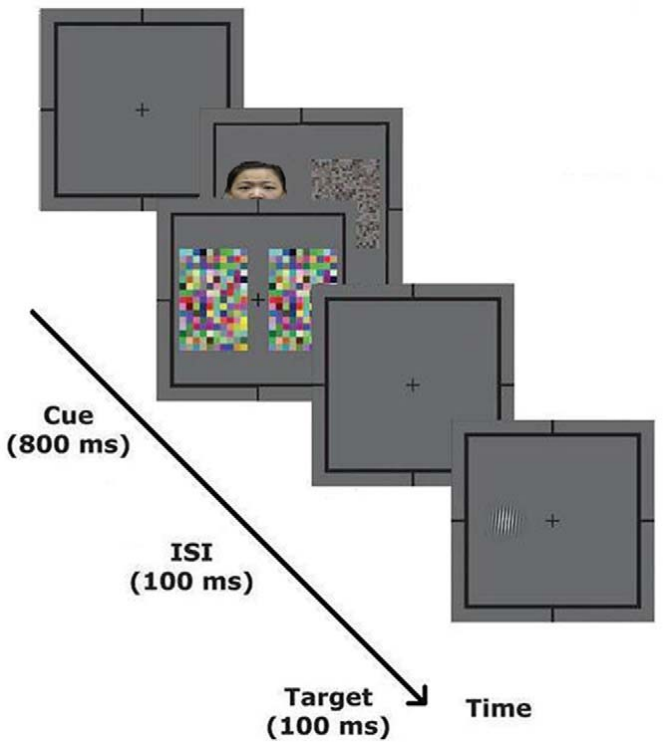
A schematic description of the experimental procedure in the invisible condition.

Stimuli were presented on a 17-inch SAMSUNG monitor (1280×1024). The two eyes' images were displayed side-by-side on the monitor and fused using a mirror stereoscope mounted on a chinrest. A frame (10.7°×10.7°) that extended beyond the outer border of the stimulus and fixation point was presented to facilitate stable convergence of the two eyes' images. The viewing distance was 40 cm. Each trial began with fixation on a central cross (0.8°×0.8°) presented to each eye. In the invisible condition, a pair of identical high contrast dynamic noise patches was presented to the observer's dominant eye and a pair of intact and scrambled images to the non-dominant eye (see Figure 1). Each image subtended 4.1°×6.2° of visual angle and was presented for 800 ms, and the horizontal distance between the centers of this pair of images was 5.8°. In this condition, observers perceived identical noise patches on both sides and were unaware of which side contained the intact or scrambled image. The visible condition was the same as the invisible condition except that the pair of dynamic noise patches that were presented to the observers' dominant eye was replaced with the same pair of intact and scrambled images that were presented to the non-dominant eye. Hence, observers could perceive the intact and scrambled images instead of the noise patches. The stimulus presentation was followed by a 100-ms interstimulus interval in which only the fixation was displayed, and then a small gabor patch (2.5°×2.5°) was presented for 100 ms as a probe in the position that either the intact or scrambled image previously occupied. The gabor patch was tilted one degree clockwise or counter-clockwise, and the participants were required to press one of two buttons to indicate their perceived orientation of the gabor patch regardless of the side of presentation (see Figure 2).

Total 256 trials were randomized across experimental conditions, including position of face image (left or right to the fixation point), position of the gabor probe (left or right to the fixation point), face emotion (fearful or happy), face gender (male or female) and visibility (visible or invisible). These trials were divided into four blocks, 64 trials for each block.

Before the experiment, participants practiced 50 trials for the invisible condition to get familiar with the experimental procedure. Those who reported seeing face images in the invisible condition were excluded from the experiment.

#### Design

For the independent variables, the between-subject variables were group (high trait anxiety vs. low trait anxiety) and gender (female vs. male). The within-subject variables were emotion (fearful vs. happy) and visibility (visible vs. invisible). The dependent variable was the orientation discrimination accuracy of the gabor patch. The working hypothesis was that if there were attentional effects (either bias or avoidance) induced by the emotional pictures as a cue, the discrimination accuracy would be increased or decreased. We quantified attentional effect as the discrimination accuracy of the gabor probe presented at the position of the intact image minus the discrimination accuracy of the gabor probe presented at the position of the scrambled image, following the method in Jiang et al. (2006) [19].

A positive value of attentional effect indicated attentional bias, which meant that attention was oriented toward emotional images, and a negative value indicated attentional avoidance, which meant that attention was oriented away from emotional images. Attentional effects were analyzed separately for the visible condition and the invisible condition, and the later one was one of the focuses of this study.

### Results

#### Visible condition

**Figure 3:**
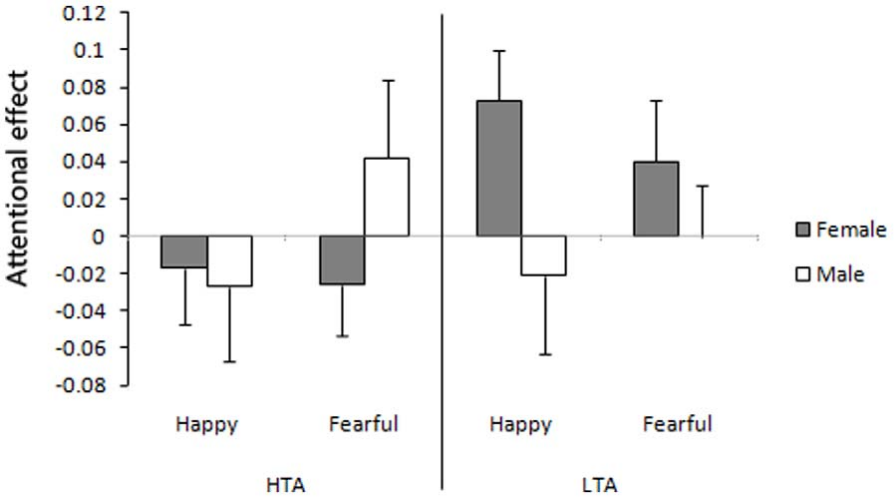
Attention bias and avoidance by happy and fearful faces in the visible condition. The results indicated no significant main effect or interaction. Error bars denote 1 SEM calculated across subjects.

Attentional effects by happy and fearful faces in HTA and LTA groups are presented in Figure 3. A 2×2×2 mixed-design ANOVA, with face emotion (happy/fearful) as within-subject variable, and anxiety state (HTA/LTA) and gender (female/male) as between-subject variables, revealed only a marginal effect for state × gender (F (1, 44) = 3.75, *p* = 0.059), no other significant main effect and interaction.

#### Invisible condition

**Figure 4:**
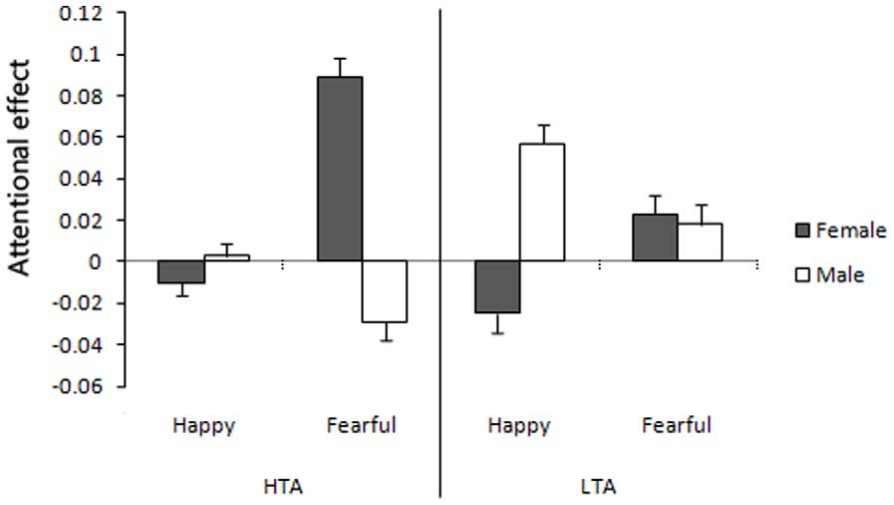
Attention bias and avoidance by happy and fearful faces in the invisible condition. The results indicated a gender difference of attentional effect induced by emotional pictures. And the gender difference of attentional effect was dependent on anxiety state. Error bars denote 1 SEM calculated across subjects.

Attentional effects by happy and fearful faces in HTA and LTA groups are presented in Figure 4.

A similar 2 (face emotion)×2 (anxiety state)×2 (gender) mixed-design ANOVA showed a significant interaction between emotion and gender (F (1, 44) = 6.59, *p* = 0.014), which indicated a gender difference of attentional effect induced by emotional pictures. The interaction between gender and anxiety state was significant (F (1, 44) = 4.77, *p* = 0.034), suggesting that the gender difference of attentional effect was dependent on anxiety state. Thus, we performed 2 (face emotion)×2 (gender) ANOVA s for the HTA and LTA groups separately. The interaction between face emotion and gender reached a significant level in the HTA group (F (1, 22) = 5.35, *p* = 0.031), but not in the LTA group (F (1, 22) = 1.89, *p* = 0.183). In addition, the HTA group also exhibited a marginally significant gender effect (F (1, 22) = 4.11, *p* = 0.055). A one sample t-test was conducted to further confirm the effect of interaction, and revealed that female participants in the HTA group showed a significant attentional bias towards fearful faces (t (11) = 2.66, *p* = 0.022). It is also worth noting that male participants in the HTA group showed a marginally significant attentional avoidance of fearful faces (t (11) = 2.01, *p* = 0.069).

## Experiment 2

In Experiment 1, we found a marginally significant attentional effect (avoidance) by fearful faces for HTA male participants in the invisible condition. It might be due to a small sample size (12 participants). Here, we conducted a second experiment employing a similar procedure with more participants. We also included neural face pictures as stimuli to examine if there was any difference between neural faces and emotional (happy or fearful) faces.

### Methods

#### Participant

The experiment was conducted in accordance with general ethical guidelines in psychology. We have obtained written informed consent from all participants and the process was approved by the ethics committee in Peking University. Participants were also recruited from the pool of 1200 college students at Peking University. 18 HTA females and 18 HTA males participated in the experiment. The range of their age was 19–26 years, and their STAI-TAI scores were in Table 2.

**Table 2:** STAI-TAI scores of female and male participants in HTA group and T-Test between two genders.

	Female	Male	t	P
HTA	52.83(9.77)	52.83(6.64)	0.00	1.00

#### Stimuli and Procedure

Neutral face images were also from the Chinese Affective Picture System (CAPS) [34] and the experimental procedure was the same as that in Experiment 1.

#### Design

A between-subject independent variable was gender (female vs. male). Within-subject independent variables were emotion (fearful vs. neutral vs. happy) and visibility (visible vs. invisible). Data were analyzed separately for the visible condition and invisible condition.

### Results

**Figure 5:**
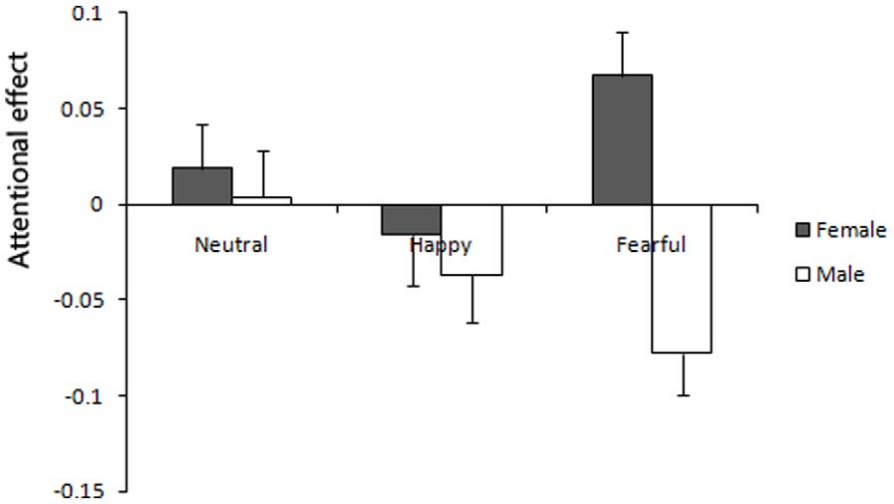
Attention bias and avoidance by neutral, happy and fearful faces in the invisible condition. Female participants exhibited attentional bias to fearful faces, while male participants exhibited attentional avoidance of fearful faces. This result supported that there was gender difference in HTA population. Additionally, we did not find attentional effects by both neutral and happy faces. Error bars denote 1 SEM calculated across subjects.

Attentional effects by neutral, happy and fearful faces in the HTA group were presented in Figure 5. A 2 (female/male)×3 (happy/neutral/fearful) mixed-design ANOVA was performed for the visible condition and invisible condition separately.

No significant effects were found in the visible condition. In the invisible condition, the interaction of gender and emotion was significant (F (2, 33) = 5.6, *p* = 0.008), and the main effect of gender was also significant (F (1, 34) = 8.62, *p* = 0.006). A one sample t-test found that, female participants exhibited attentional bias to fearful faces (t (17) = 2.89, *p* = 0.01), while male participants exhibited attentional avoidance of fearful faces (t (17) = −3.75, *p* = 0.002). This result supported that there was gender difference in HTA population. Additionally, we did not find attentional effects by both neutral and happy faces (see Figure 5).

## Discussion

Using binocular suppression to render face images invisible, we found that invisible fearful faces could alter the distribution of spatial attention in HTA individuals. The attentional effect was gender-dependent. Specifically, HTA males showed attentional avoidance of invisible fearful faces, but HTA females showed attentional bias towards them. No significant attentional effect was found in the visible condition, in LTA individuals, and with neutral and happy face images.

Consistent with previous studies [5], [12], [35], we did not find attentional avoidance of fearful faces in the HTA group in the visible condition. Such a reliable observation across 800 ms, 1250 ms and 1500 ms presentation duration demonstrates that the null effect is not likely to be an artifact and this observation cannot be fully explained by the “vigilance-avoidance” model proposed by Mogg and Bradley (1998). On the other hand, the attenitonal effect in the invisible condition and its gender difference support our hypotheses and make us to re-think about cognitive processes at unconscious level.

In the invisible condition, HTA male participants exhibited attention avoidance of fearful faces, which can be considered to have some positive values. Recent models about attention to threat [7], [8], [36] have emphasized the adaptive value of strategic attentional avoidance in some situations. For example, when some stimuli need not to be processed immediately, attention avoidance could be a good strategy to complete current tasks [8], or regulate mood by avoid processing negative information [37]. HTA female participants exhibited attention bias to fearful faces. The bias may not indicate attention shift to the threatening images because of their long presentation. Instead, it might reflect female participants' difficulty to disengage their attention from threatening stimuli. Since the shift of spatial attention could be operated at a fine temporal scale (e.g. 200–300 ms, see [38]), the 800 ms presentation time in our study is sufficient for participants to move attention towards and away from non-prefered stimuli. The disengage difficulty in HTA female participants may reflect their excessive processing in threatened materials [39]. This result is consistent with a previous study that women may tend to overestimate the potential of threat, and are more anxiety sensitive than men [40]. This might have some implications for clinical practice. MacLeod and Hagan [41] had females who were waiting for gynecology procedure to do a masked Stroop task and told some of them that they have been diagnosed cervicitis. They showed that attentional bias induced by subliminal stimuli could predict the following emotional collapse. Thus, subliminal attentional bias could reflect one's vulnerability to stress. The HTA females in our study may be vulnerable to fearful faces, so that they could not direct their attention away from the negative information.

Our study emphasizes two important issues in psychopathological researches. One is consciousness manipulation, the other is gender difference. Previous studies [8], [42], [43] have tried different presentation durations to manipulate consciousness, some of which were combined with backward masking. Cognitive information processing at conscious level typically involves both bottom-up and top-down processes. On the other hand, unconscious processing is usually considered to a bottom-up process, which might reflect an instinctive process without top-down cognitive controls [44]. Measuring emotional processing at conscious level usually suffers the cognitive inferences (e.g. strategies) from top-down processes, which might prevent a direct measure of the instinctive process. Using invisible stimuli is a feasible way to overcome this difficulty. The finding of attentional effect only in the invisible condition supports our view. What' more, from the psychodynamic perspective, our result may also reflect the different unconscious effect of previous psychological experiences on HTA and LTA individuals. It should also be noted that binocular suppression has many advantages for studying unconscious emotional processing (e.g. the long and complete suppression of stimuli out of awareness, see the review by Kim and Blake [45]). The technique has been used to accurately predict sexual orientation [19]. It is worthwhile to apply this technique to other emotional researches.

We demonstrated the existence of gender difference in anxiety population and suggested the importance of balancing participants' gender in future studies. Previous studies used anxious participants with different ratios of genders, which generated distinctive conclusions. Our study adopted equal numbers of female and male participants and found significant, but different, attentional effects for each gender. Future studies should consider gender difference as an important factor in anxiety research.

In conclusion, we found attentional effects induced by fearful faces at unconscious level, and the effects were distinct for male and female participants. These findings may contribute to our understanding of gender difference in anxiety disorder.
